# 

**Published:** 1888-01

**Authors:** 


					﻿Vol VIIL
JANUARY, 1888.
NO. 1.
THE
pOMCEOPATHIC pHY£lClA]fl,
A Monthly Journal of Homoeopathic Materia Medica
and Clinical Medicine.
“ He is a freeman whom truth makes free, and all are slaves beside.”
” Seek the Truth : come whence it may, cost what it will.”
EDITED AND PUBLISHED BY
EDMUND J, LEE, M. £>.,
AND
WALTER Nl. JAMES, Ml. D.
PHILADELPHIA:
No. 1123 SPRUCE STREET.
r. O 1ST ±> O IT :
ALFRED HEATH & CO., Sole Agents FOB Great Britain,
' 114 Ebury Street, Eatou Square.
Yearly Subscription, $2.50, invariably in advance. Single numbers, 25 cts.
Entered at the Philadelphia Poet-Office as Second-Class Mall Matter.
				

